# Prevalence and predictive risk factors of hypertension in patients hospitalized in Kamenge Military hospital and Kamenge University teaching hospital in 2019: A fixed effect modelling study in Burundi

**DOI:** 10.1371/journal.pone.0260225

**Published:** 2021-12-13

**Authors:** Arnaud Iradukunda, Emmanuel Nene Odjidja, Stephane Karl Ndayishima, Egide Ngendakumana, Gabin Pacifique Ndayishimiye, Darlene Sinarinzi, Cheilla Izere, Nestor Ntakaburimvo, Arlene Akimana

**Affiliations:** 1 Department of Medicine, University of Burundi, Bujumbura, Burundi; 2 Department of Statistics, Lake Tanganyika University, Mutanga, Burundi; 3 Royal Society of Tropical Medicine and Hygiene, London, United Kingdom; 4 Department of Research and Innovation, ARNECH Research and Consulting Office, Bujumbura, Burundi; 5 Department of Medicine, School of Clinical Sciences, Monash University, Clayton, VIC, Australia; 6 Departemend de Medecine, université Paris-Est-Créteil-Val-de-Marne, Créteil, France; 7 Department of Applied Econometrics, University of Lille, Villeneuve-d’Ascq, France; 8 Departement de Suivie-Evaluation des projects, Instututs Sciences Campus du Centre, Ouagadougou, Burkina-Fasso; 9 Department of Computer Mathematics, Clermont Auvergne University, Clermont-Ferrand, France; 10 Institut Universitaire des Sciences de la Santé et du Développement, Bujumbura, Burundi; 11 Department of Public Health, Aix-Marseille University, Marseille, France; Faculty of Medicine, SERBIA

## Abstract

**Introduction:**

Hypertension is a major threat to public health globally. Especially in sub-Saharan African countries, this coexists with high burden of other infectious diseases, creating a complex public health situation which is difficult to address. Tackling this will require targeted public health intervention based on evidence that well defines the at risk population. In this study, using retrospective data from two referral hospitals in Burundi, we model the risk factors of hypertension in Burundi.

**Materials and methods:**

Retrospective data of a sample of 353 randomly selected from a population of 4,380 patients admitted in 2019 in two referral hospitals in Burundi: Military and University teaching hospital of Kamenge. The predictive risk factors were carried out by fixed effect logistic regression. Model performance was assessed with Area under Curve (AUC) method. Model was internally validated using bootstrapping method with 2000 replications. Both data processing and data analysis were done using R software.

**Results:**

Overall, 16.7% of the patients were found to be hypertensive. This study didn’t showed any significant difference of hypertension’s prevalences among women (16%) and men (17.7%). After adjustment of the model for cofounding covariates, associated risk factors found were advanced age (40–59 years) and above 60 years, high education level, chronic kidney failure, high body mass index, familial history of hypertension. In absence of these highlighted risk factors, the risk of hypertension occurrence was about 2 per 1000 persons. This probability is more than 90% in patients with more than three risk factors.

**Conclusion:**

The relatively high prevalence and associated risk factors of hypertension in Burundi raises a call for concern especially in this context where there exist an equally high burden of infectious diseases, other chronic diseases including chronic malnutrition. Targeting interventions based on these identified risk factors will allow judicious channel of resources and effective public health planning.

## Introduction

Hypertension corresponds to a permanently raised blood pressure in arteries and arterioles [1]. It is defined as a systolic blood pressure equal or above 140 mmHg and /or a diastolic blood pressure above 90 mmHg [1, 2]. Hypertension is a threat to global public health [3] as it tires vessels, the heart and causes damage to artery walls [4, 5]. It is a major risk factor for cardiovascular diseases [6]with high morbidity and mortality rate [6]. If not identified and treated early, arterial hypertension may result in serious complications including strokes, coronary artery, kidney and hypertensive heart diseases [1, 7]. These complications are among the leading causes of mortality in the world. Approximately, cardiovascular diseases account for 17.8 million death in 2017 [8, 9], nearly 1/3 of total. More than three quarters were in low and middle-income, countries (LMICs) [9]. Hypertension complications, cardiovascular diseases account 9.4 million (52.8%) every year [10]. Hypertension is responsible for 45% deaths due to heart disease and 51% stroke related deaths [11, 12]. Premature death and health care expenditure for treatments due the hypertension puts an economic toll on families and pushes many into poverty [13, 14]. At the macro level, these high expenses and human losses significantly impacts on economic growth and reduces productivity [15, 16].

The prevalence of hypertension in adults was 40% only in 2015, with an estimated 1.13 billion people living with different forms of hypertension [17, 18]. Data from the World Health Organisation Global Health Observatory Repository [19] found the highest prevalence of hypertension in the Africa region (46%) followed by the America (35%) and other regions, majority of whom, were undiagnosed and untreated [20, 21]. In sub-Saharan Africa (SSA), as other settings, hypertension has been associated with lifestyles, diets, physical inactivity urbanization and socio economic status [22]. More than 125 million people with hypertension are expected by the year 2025 in SSA alone [23]. By year 2030, hypertension and other non-communicable diseases are projected to surpass communicable diseases as the top of mortality causes on the continent [24]. From 2011 to 2025, the cumulative lost output with non-communicable diseases is projected to be US$7.28 trillion in LMICs which is approximately a loss of US$500 billion per year [25]. Cardiovascular diseases including hypertension account for nearly half this cost [26]. Despite this, SSA faces a major problem of early screening, timely treatment and control of hypertension [27, 28]. Yet, studies to understand the epidemiology and associated risk factors of hypertension in the context of Burundi are lacking, prompting the conduct of this study. Therefore, in this study, we determined the overall prevalence of hypertension. We also evaluated its predictive risk factors and its occurrence probability based on risk factors. Knowing these factors could support effective public health planning and facilitate policy makers to formulate plausible policies towards the fight against hypertension and its complications.

## Materials and methods

### Ethics statement

This investigation is in accordance with Helsinki’s Declaration and approved by the institutional Research Ethics committees. Written permissions were acquired from ethical committee of the University teaching hospital of Kamenge and Kamenge military hospital to use retrospective data for this study. In effort to secure identity of patients, patient’s information were anonymised and replaced by a unique codes. Data are used for the unique objective of this study and will be destroyed after paper publication.

### Study description

We employed a cross-sectional study. The study was conducted in two tertiary reference hospitals of Burundi: University teaching hospital of Kamenge and Kamenge Military hospital in different departments. Patients were hospitalized for different medicals diseases including infectious diseases, cardiovascular diseases and others. Based on socio-demographic and clinical characteristics associated with hypertension, the study used data from internal medicine services with or not haemodialysis sessions (for patients with kidney failure) where majority of patients with hypertension and others cardiovascular diseases are admitted Data were collected among patients hospitalized from January 2019 to December 2019 in these services. These hospital were firstly chosen because they are ranked as tertiary national reference and their receive patients from all over the country. Secondly, one is private hospital and another a public hospital.

### Sampling methods

This study targeted a population of 4380 patients hospitalized in internal medicine services and intensive care unit (with chronic kidney failure) during the whole study period in the both hospitals. Majority of these patients are hospitalized there because this areas are located together with haemodialysis unit. Among them, a sample size of 353 patients have been selected. The inclusion probability were the same for patients admitted in the same service of the selected hospital and was calculated as ratio of admitted patients in the service over all admissions of all services. Patients were randomly selected in the service using their specific identifiers of admission. The minimal sample size was calculated based on the quintile of the normal distribution with at 95% of confidence (1.96), the population size, the prevalence of hypertension and the acceptable margin (5%) [28]. As the prevalence of hypertension is unknown in Burundi, a value of *p* = 0.5 was selected. According to these parameters, the minimal size of the sample was 353 patients. Basically, a respondent was selected if he had of the following criteria: Admitted in targeted service in the period of 2019, measured diastolic and systolic blood pressure three times.

Normally, after resting in a quiet environment for 5–10 minutes, the patient took a sitting position with his legs naturally flat and his right hand placed at heart height. An Omron HEM-705P arm-type electronic sphygmomanometer were used to measure the systolic blood pressure, diastolic blood pressure and resting heart rate of the brachial artery of the right upper arm at least twice, with an interval of no less than two minutes. The average of the two readings was taken.

### Data collection

In total, a population of 4380 patients stratified in 2 groups in both hospitals were targeted. A random sampling method have been used with proportional allocation to the admitted patients by service and by hospital. Data were collected using a standard-structured questionnaires based on hospital record. We collected socio-demographics, anthropometrics and clinical characteristics as summarized in **Table 1**. All patients with hypertension appeared before chronic kidney failure were excluded in the study. In this context, chronic kidney failure is predictor of hypertension.

**Table 1:** Descriptive analysis of patients’ characteristics.

Individual characteristics	Category	N	H^-^	H^+^	P+	*95% CI of P+*
Sex	Men	206	173	33	16.0	[11.3–21.8]
	Women	147	121	26	17.7	[11.9–24.8]
Age group	15–39 years	138	135	3	2.2	[0.5–6.2]
	40–59 years	131	103	28	21.4	[14.7–29.4]
	≥60 years	84	56	28	33.3	[23.4–44.5]
Marital status	Married	89	81	8	9.0	[39.0–16.9]
	Unmarried	264	213	51	19.3	[14.7–24.6]
Educational level	Primary or less	137	123	14	10.2	[5.7–16.6]
	Secondary	165	135	30	18.2	[12.6–24.9]
	University	51	36	15	29.4	[17.5–43.8]
Alcohol	No	173	145	28	16.2	[11.1–22.5]
	Yes	180	149	31	17.2	[12.0–23.6]
Smoking	No	325	274	51	15.7	[11.9–20.1]
	Yes	28	20	8	28.6	[13.2–48.7]
Existing cardiovascular impairment	No	28	24	4	14.3	[4.0–32.7]
	Yes	325	270	55	16.9	[13.0–21.5]
Family History	No	289	262	27	9.3	[6.3–13.3]
with Hypertension	Yes	64	32	32	50.0	[37.2–62.7]
Diabetes	No	273	247	26	9.5	[6.3–13.6]
	Yes	80	47	33	41.3	[30.4–52.8]
Chronic kidney failure	No	169	164	5	3.0	[1.0–6.7]
	Yes	184	130	54	29.3	[22.9–36.5]
Body mass index	Underweight and normal	270	236	34	12.6	[8.9–17.3]
	Overweight	60	44	16	26.7	[16.5–39.9]
	Obesity	23	14	9	39.1	[20.5–61.2]
Overweight	No	271	237	34	12.5	[8.9–17.1]
	Yes	82	57	25	30.5	[20.8–41.7]
**Total**		**353**	**294**	**59**	**16.7**	**[15.6–25.1]**

N: Category’s total, H^-^: Normotensive people, H^+^: Hypertensive people, P^+^: Hypertensive people proportion

### Outcome and independent variables

In this study, hypertension was considered as outcome variable and was defined as systolic blood pressure ≥ 140 mmHg and/or diastolic blood pressure ≥ 90 mmHg obtained after three successive measurements or patients on hypertension’s treatment. Pre-hypertension was defined as BP levels of 120 to 139/80 to 89 mm Hg [29]. Body mass index (BMI) was calculated as the weight in kilograms to square of height in meters and was categorized in into; underweight (BMI<18.5 kg/m^2^), normal (BMI: 18.5–24.9 kg/m^2^), overweight (BMI: 25.0–29.9 kg/m^2^) and obesity (BMI ≥ 30 kg/m^2^). During data analysis, BMI was categorized in three clusters (Under and normal weight, overweight, obesity). Others independents variables were classified as age group (in years) (15–39 years, 40–60 years; ≥60) sex (Men, Women), residence (Urban, Rural), educational level (Primary or less, Secondary, University), alcohol consumption (Yes/No), smoking (Yes/No), diabetes (Yes/No), existing cardiovascular impairment (Yes/No), familial history of hypertension (Yes/No) and chronic kidney failure (Yes/No). As chronic kidney failure can be predictor or consequence of hypertension, only patients with history of kidney failure before hypertension were included in the study.

## Statistical analysis

Data analysis were undertaken in different steps: descriptive analysis, binary logistic modelling with fixed effects, power predictive evaluation of final (saturated) model and probabilities prediction. Hypertension associated risk factors were assessed using univariate and multivariate logistic regressions. We calculated the odds ratios (ORs) at 95% confidence level for each covariate to identify predictors of hypertension. Significant variables on 15% threshold were used in multivariable logistic modelling to determine a combined effect on the outcome. The likelihood ration test, the score test and the Wald test were used to determine significance of independent variables on the outcome [30]. A threshold of 5% have been used. To select the best model for this study, we use backward method with parsimony principle [31]. The Akaike Information Criterion (AIC) based on adjustment were used [32]. The best model is one with low AIC value.

The relevance of the final model to make prediction was assessed by Pearson residuals test. The Receiving Operating characteristics (ROC) and Area under Curve (AUC) were respectively used to compare and evaluate performance and predictive power of the model. Furthermore, the ROC was used to determine the discriminatory performance of the model, determining the false positive and false negative rates. The Mann Whitney statistics method showed that the two distributions were offset: normotensive people had an average higher scores than hypertensive people. Each individual’s score was ranked in ascending order. Thus, the AUC which determined the number of observations accurately predicted was calculated based on the number of normotensive patients, the number of hypertensive patients and the ranks of normotensive patients. If AUC = 0.5 no discrimination, 0.7≤AUC≤0.8 acceptable discrimination, 0.8≤AUC<0.9 excellent discrimination, AUC≥0.9 exceptional discrimination. To calculate the error of predictions, the cross validation with K -Fold method have been used. We used 10-folds. In validating internal performance of the model, a bootstrapping procedure with 2000 replications was done. Statistical significance was considered when p-value ≤ 0.05.

Influents points of the model were assessed using Hoaglin and Welsh criterion, methods based on model’s parameters and the sample size. The adjustment used was based on the residuals test. The Kolmogorov test was used to test the model adequation. For these test, a threshold of 5% was used. Then, the foreign, rpart and forest model packages were used to carry out results in this study [33]. Data processing and data analysis were done using R.3.5.0 software.

## Results

In this study, the overall prevalence of hypertension was 16.7% (**Table 1**). Comparatively, men’s and in women’s prevalence were respectively 16.0% and 17.7% with insignificant difference (X^2^ = 0.0725, df = 1, p = 0.788). This prevalence was 2 times higher in overweight patients than normal and underweight patients and 3 times higher in diabetic patients than no diabetics patients. The pre hypertension was observed in 73.4% of patients. The high proportions above the overall prevalence were observed in people with existing cardiovascular impairment, married, patients aged between 40–60 years or 60 years and above and over, patients with chronic kidney failure, men patients, smokers patients, obese patients and patients with secondary or university level (**Table 1**). The table below showed descriptive analyses of patients’ characteristics.

### Logistic regression modelling of predictive risk factors of hypertension

The Table 2 showed the univariate logistic regressions where hypertension was modelled by each explanatory variable.

**Table 2:** Univariates logistic regressions of hypertension’s factors.

Variables	Category	OR	95% CI	p
Educational	Primary or less	Reference		
level	Secondary	1.9	[1.0–3.9]	0.054
	University	3.7	[1.6–8.4]	0.002
Smoking	No	Reference		
	Yes	2.1	[0.9–05.0]	0.085
Diabetes	No	Reference		
	Yes	6.7	[3.7–12.3]	<0.001
Age group	15–39 years	Reference		
	40–59 years	12.2	[4.2–52.1]	<0.001
	≥60 years	22.5	[17.6–96.8]	<0.001
Currently working	No	Reference		
	Yes	0.9	[0.6–1.7]	0.944
Residence	Rural	Reference		
	Urban	1.1	[0.6–1.8]	0.868
Body mass index	Underweight and normal	Reference		
	Overweight	2.5	[1.3–4.9]	0.007
	Obesity	4.5	[1.7–10.9]	0.001
Sex	Women	Reference		
	Men	0.8	[0.6–1.9]	0.451
Marital status	Unmarried	Reference		
	Married	2.4	[1.2–5.7]	0.027
Existing cardiovascular impairment	No	Reference		
	Yes	1.2	[0.5–4.3]	0.720
Alcohol	No	Reference		
	Yes	1.1	[0.6–1.9]	0.794
Chronic Kidney	No	Reference		
failure	Yes	13.6	[5.8–39.9]	<0.001
Familial History	No	Reference		
of Hypertension	Yes	9.7	[5.2–18.4]	<0.001

OR: Odd ratio, CI: Confident interval, p: p-value.

Eight variables were significantly associated with hypertension in univariate regression (**Table 2**). Those variables were Educational level{University (OR: 3.7, 95% CI: 1.6–8.4, p<0.002); Secondary (OR: 1.9, 95% CI: 1.1–3.9, p: 0.045), Diabetes (OR: 6.7, 95% CI: 3.37–12.3, p<0.001), Age {(40–59 years (OR: 12.2, 95% CI: 4.2–22.1, p<0.001); 60 and above (22.5, 95% CI: 7.6–26.8, <0.001)}, Body mass index {Overweight (OR: 2.5, 95% CI: 1.3, 4.9, p: 0.007); Obesity (OR: 4.5, 95% CI: 1.7–10.9, p: 0.001)}, Marital status (OR: 2.4, 95% CI: 1.2–5.7, p: 0.027), Chronic kidney failure (OR: 13.6, 95% CI: 5.8–19.9, p<0.001) and Family hypertension history (OR: 9.7, 95% CI: 5.2–18.4, p<0.001). All these variables were introduced in a multivariate analysis. In addition, other relevant variables were introduced in the multivariate model as well as sex, smoking, alcohol and existing cardiovascular impairment. The identification of variables based on AIC and backward selection methods showed six variables significantly associated with hypertension.

Then, the equation of saturated model derived from the above multivariate model include the Educational level, Body mass index, Chronic kidney failure and familial history of hypertension. After controlling the cofounding variables as indicated above, adjusted odds ratio and their lower and upper bound along with a corresponding p-value were derived. Table 4 detailed these results.

The Table 3 showed that advanced age and education level were the most associated risk factor of hypertension. Chronic kidney failure, overweight, obesity, familial hypertension history were significantly associated with hypertension. Patients with secondary level had near five times higher (AOR: 4.9, 95% CI: 2.1–12.4, p<0.001) the risk of hypertension occurrence than patients with primary or less educational level. That risk was more than 8 times higher (AOR: 8.6, 95% CI: 2.9–27.3, p<0.001) in patients with university level than patients with none or primary level. Even if smokers had more than two times high the risk (AOR: 2.9, 95% CI: 0.8–9.3, p: 0.073) to become hypertensive than non-smokers, tobacco consumption were not significantly associated with hypertension. Tobacco consumption remained in saturated model because it minimized the AIC criterion than the model with no tobacco use as explanatory variable. Patients aged between 40–59 years had more than 5 times (AOR: 5.9, 95% CI: 1.9–26.8, p: 0.007) the risk to become hypertensive than patients aged under 40 years. That risk was more than 12 times higher (AOR: 12.9, 95% CI: 3.4–65.8, p<0.001) in patients aged over 60 years than young patients under 40 years. Patients with family history of hypertension had near three times higher (AOR: 2.9, 95% CI: 1.3–6.7, p: 0.007) the risk to become hypertensive than patients with non familial hypertension history. This risk was more than two times (AOR: 2.5, 95% CI: 1.1–5.7, p: 0.037) in overweight patients than normal and underweight patients. That risk of hypertension was more than 3 times higher (OR: 3.7, 95% CI: 1.2–11.5, p: 0.023) among obese patients than normal and underweight. Patients with chronic kidney failure had approximately 5 times (AOR: 4.9, 95% CI: 1.8–15.6, p: 0.003) the risk of hypertension than normal patients. The Wald test (X^2^ = 86.7, df = 8, p<0.001) rejected the null hypothesis and therefore to confirm the alternative hypothesis stating that there is at least one estimate significantly different to zero. This suggests an overall significance of the model. Pearson residuals test of (X^2^ = 266.17, df = 344, p = 0.99) was done and showed the model was well adjusted on the observations. A McFadden statistic (R^2^: 0.4) also indicated that this model had a good fit.

**Table 3:** Results of multivariate logistic regression.

Variables	Category	N	H^-^	H^+^	AOR	95% CI	p
Education level	Primary or less	137	123	14	Reference		
	Secondary	165	130	35	4.6	[1.9–11.5]	<0.001
	University	51	36	15	8.5	[2.9–27.2]	<0.001
Smoking	No	325	274	51	Reference		
	Yes	28	20	8	2.9	[0.9–9.3]	0.073
Age group	15–39 years	138	135	3	Reference		
	40–59 years	131	103	28	5.9	[1.8–26.8]	0.007
	≥60 years	84	56	28	12.9	[3.4–65.8]	<0.001
Body mass	Underweight and normal	270	236	34	Reference		
index	Overweight	60	44	16	2.5	[1.1–5.7]	0.037
	Obesity	23	14	9	3.7	[1.2–11.5]	0.023
CKF	No	169	164	5	Reference		
	Yes	184	130	54	4.9	[1.8–15.6]	0.003
FHH	No	289	262	27	Reference		
	Yes	64	32	32	2.9	[1.3–6.7]	0.014

H^-:^ Normotensive people, H^+^: Hypertensive people, AOR: Adjusted Odd ratio, CI: Confidence interval, p: p-value, CKF: Chronic kidney failure, FHH: Familial history of hypertension.

**Fig 1:**
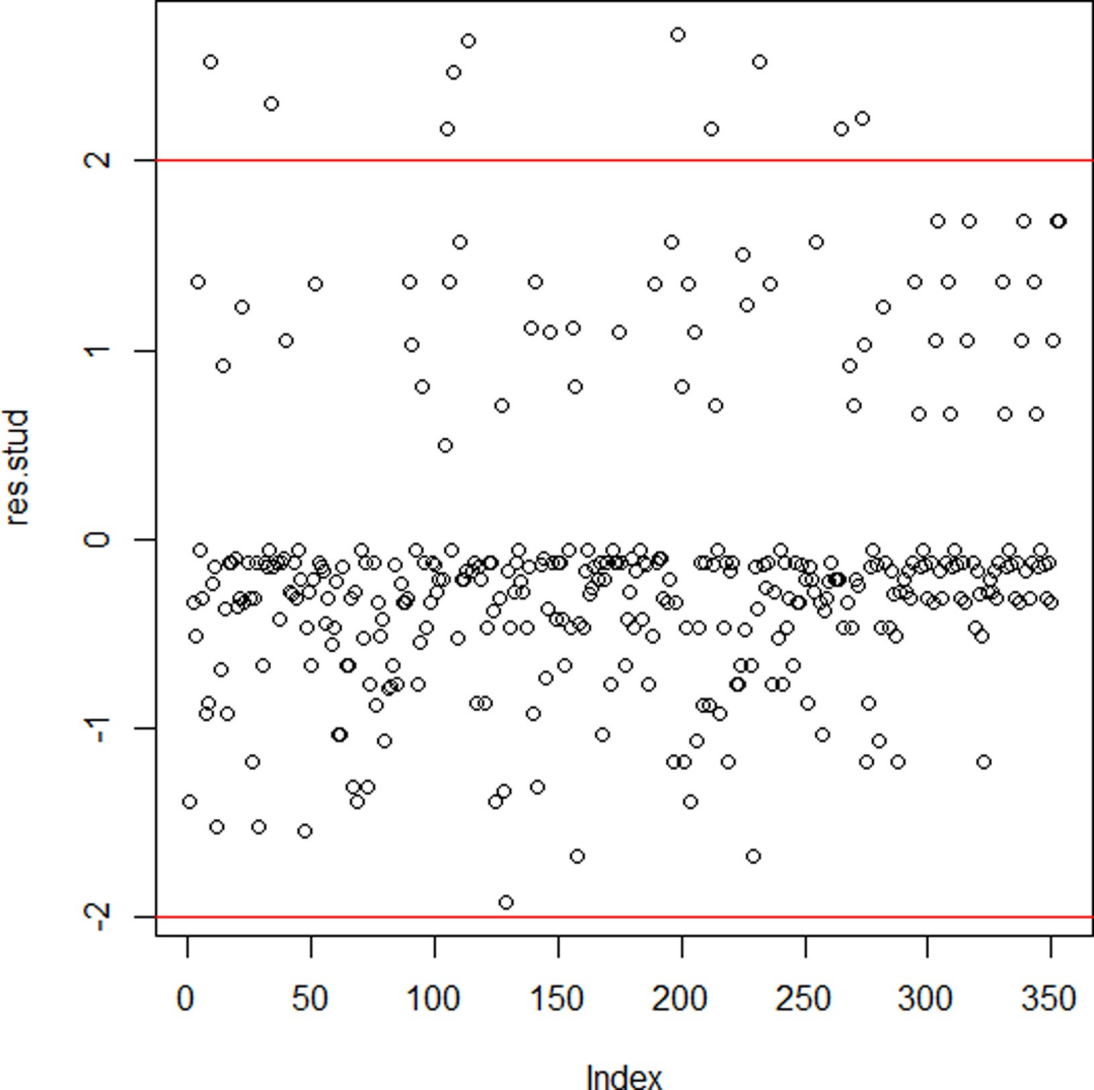
Studentized residuals.

**Fig 2:**
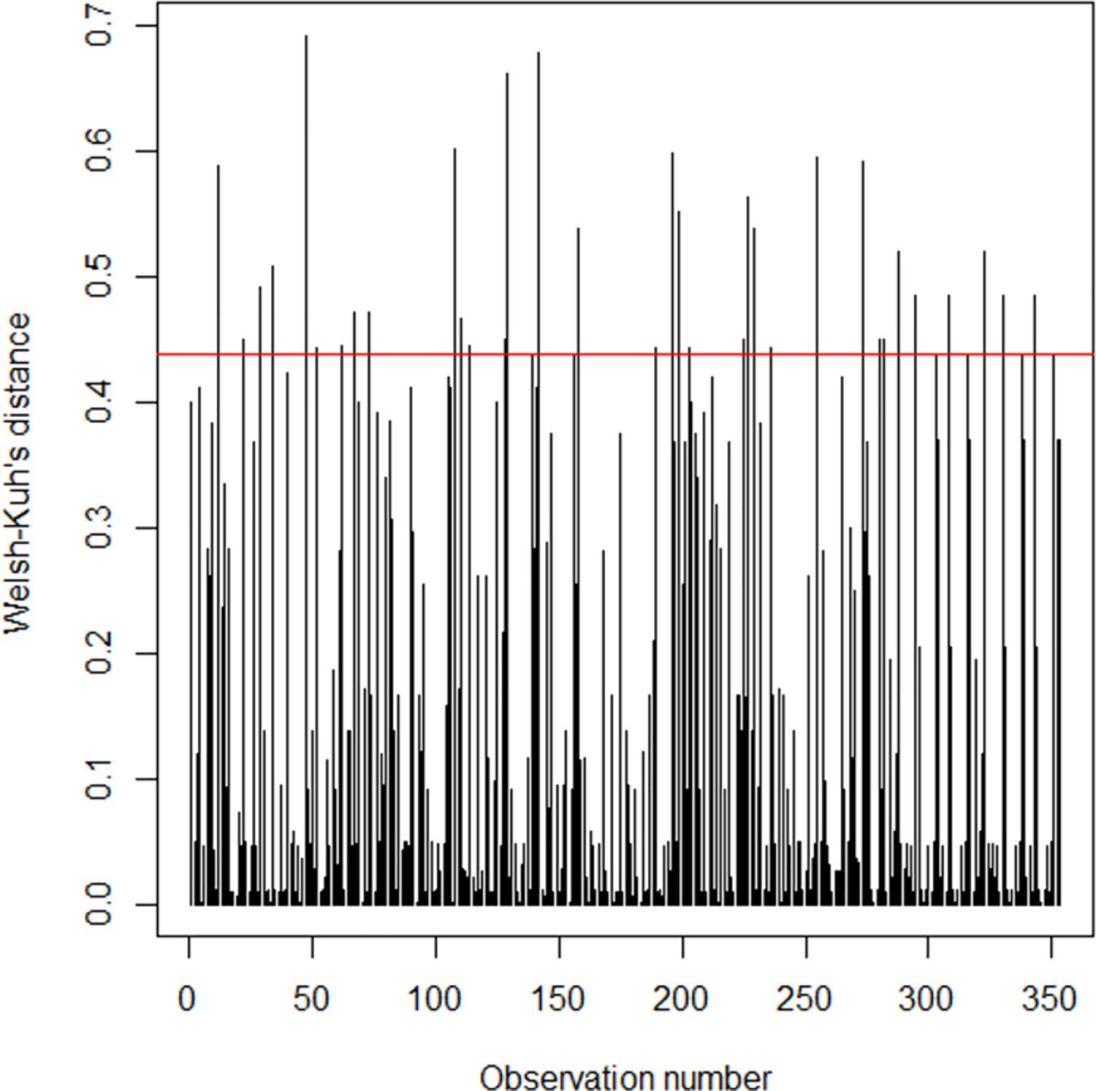
Welsh-Kuh’s distance.

The influential points’ analysis based on Hoaglin and Welsh criterion showed that only 9 points were influential. Also, Cook’s distance showed that only 3 points (108, 114 and 199) were outliers, which means the influential points were not numerous. Studentized residues analysis (Fig 1) showed that 97% (343/353) were between -2 and 2. Observations with residues (Fig 2) greater than 2 were ten (9, 34, 105, 108, 114, 199, 212, 232, 265, 273). Then, any observation with studentized residue were less than -2, indicating that the number of outliers were negligible.

### Cross validation and probabilities predictions

**Fig 3:**
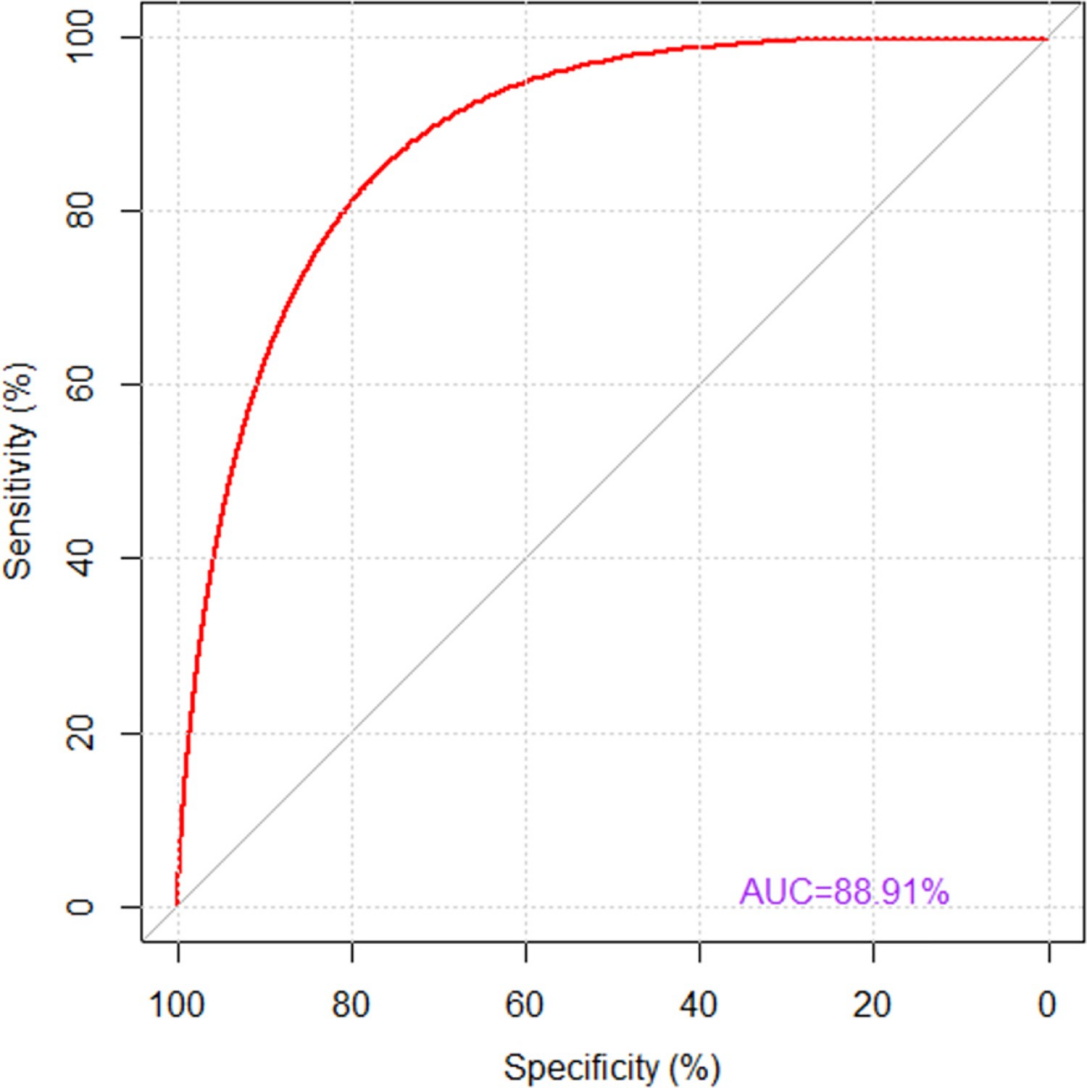
Area under curve.

The Fig 3 showed the ROC curve and area under curve (AUC). A bootstrap method, with 2000 replications, was used to determine an AUC of 88.6% (95% CI: 84.1%-92.3%) which suggested an excellent discrimination (Fig 3). This implies that saturated model had an excellent predictive power and probabilities accurately determine patients with hypertension based on identified characteristics. In supervised learning, the resubstitution error rate was 13.3%. This error rare is supposed to indicate the performance of the model when it is employed on the population. However, we know, being estimated on learning data, it was biased, and under estimating true error rate using cross-validation method which is the best quality estimator than the substitution method. The cross-validation error rate was 16.1%.

The table showed the predicted probabilities of becoming hypertensive based on different scenarios of having either a risk factor or a combination of risk factors. The first individual was the one without risk factors. He was considered as the reference individual. Sixteen predictions were generated from reference individual to whom with all hypertension risk factors. The table below showed increasing probabilities predictions from zero factors to all factors.

In absence of the risk factors highlighted above, the reference individual have 2 per 1000 the chance to become hypertensive. This probability goes from simple to more than double among smokers and 6 times higher among adult patients aged between 40 and 60 years. Then, no smokers patients with normal weight, aged between 40 and 59 years and the young obese patients (19–40 years) had probability to become hypertensive between 12% and 15%. Comparatively to the reference patient, this probability was more than 40 times higher among patients aged between 60 years and over. If the individual aged between 40 and 60 was also smoker, his probability to become hypertensive increased near 15 times (29.1%). If that patient was overweight, that probability increased from 29.1% to 78.6%. Furthermore, if the overweight patients aged between 60 and over were also smoked, their chance to develop hypertension was 99.7%. That probability was very high among patients with coexistence of more than three risk factors (99%).

The highest probability was observed among patients who were at the same time with university education level smokers, chronic kidney failure and born in the hypertensive family. The 11^th^, 12^th^, 13^th^, 14^th^, 15^th^ and 16^th^ patient were respectively at 99.7%, 99.9%, 99.9%, 99.9% and 100% risk of hypertension (**Table 4**).

**Table 4:** Predicted probabilities.

Ind.	Education level	Smoking	Age group	BMI	CKF	FHH	p
1*	Primary or less	no	15–39 years	Normal	no	no	0.002
2	Primary or less	**yes**	15–39 years	Normal	no	no	0.005
3	Primary or less	no	40–59 years	Normal	no	no	0.124
4	Primary or less	no	≥60 years	Normal	no	no	0.916
5	Primary or less	no	15–39 years	Overweight	no	no	0.016
6	Primary or less	no	15–39 years	Obesity	no	no	0.131
7	Primary or less	no	15–39 years	Normal	**yes**	no	0.005
8	Primary or less	no	15–39 years	Normal	no	**yes**	0.009
9	Primary or less	**yes**	40–59 years	Normal	no	no	0.291
10	Primary or less	**yes**	40–59 years	Overweight	no	no	0.789
11	Primary or less	**yes**	≥60 years	Overweight	no	no	0.997
12	Primary or less	**no**	≥60 years	Overweight	**yes**	**yes**	0.999
13	Secondary	**yes**	40–59 years	Obesity	**yes**	**yes**	0.999
14	Secondary	**yes**	≥60 years	Obesity	**yes**	**yes**	0.999
15	University	**yes**	≥60 years	Overweight	**yes**	**yes**	0.999
16	University	**yes**	≥60 years	Obesity	**yes**	**yes**	≈1,00

Ind.: individual, BMI: Body Mass Index, CKF: Chronic Kidney Failure, FHH: Familial hypertension

*Reference individual, p: probability of becoming hypertensive based on combination of risk factors.

## Discussion

In this study, we determined the prevalence of hypertension, identified principal predictive risk factors of hypertension and predictive probabilities to become hypertensive based on risk factors. Overall, the prevalence of hypertension was relatively high Considering women only, the prevalence was also high. That prevalence was similar to results of a recent study conducted in Lesotho which showed a relatively high prevalence (17.3%) among women [34]. These results were also in the line with previous study recently conducted in US among adults which showed a high prevalence among men than woman [35]. In another study conducted in Saudi Arabia, the prevalence was 15.2% among those aged 15 years old and above had different levels of hypertension [36].

This study did not showed the significant difference of the prevalence between women and men [36]. This finding was consistent with a studies conducted in Benin where there was no significant difference between men [37]. The highest prevalence of hypertension observed in diabetic patients and the lowest in young patients aged under 40 years [37]. This study showed that the hypertension’s prevalence was less than SSA’s hypertension prevalence. The pre hypertension was estimated on high proportion.

Literature on marital status and hypertension was inconclusive and mostly compared never married to currently married persons [38]. In congruence to this, our study did not show association between marital status and hypertension [38]. These findings were contrary to what had previously reported on this association [39]. After adjusting hypertension on other covariates using logistic regression model, high educational level, smoking, advanced age, overweight, obesity, chronic kidney failure and familial history of hypertension were significantly associated with hypertension. Similar findings were found in a previous study conducted in Malawi, Kenya and Bangladesh which showed that that the factors associated with hypertension were overweight, smoking, education level and older age [40–42]. Similar findings were also reported in recent study conducted in Nepal which showed that being overweight, obese and with hypertension’s family history were associated with hypertension [43]. The association between advanced age and high risk of hypertension could be due to the biological effects of increased arterial resistance which increases with old age [44]. This study didn’t find the association between residence and marital status. Furthermore, as in this study, alcohol was also not associated with hypertension. These findings are in line with results reported in two studies conducted in Benin and some Europeans countries [34, 37].

This study showed that predicted probabilities to become hypertensive was low in young patients aged under 40 years. High probabilities were observed in patients with advanced age. It was also observed in patients with coexistence of risk factors. The highest probabilities (≥90%) were observed among old patients with at least two additional risk factors.

One strength of our study was the ability to study hypertensive and normotensive people at the same time, combining descriptive and inferential analysis (logistic regression with fixed effects, Wald test, deviance test) to build the ROC curve. Other strengths of the study were the ability to estimate area under curve, to build bootstrap AUC interval confidence using Bootstrap method with 2000 replicates, model’s residuals analysis using Welsh-Kuh’s distance, predicting probabilities of becoming hypertensive given a combination of risk factors. Lastly, even if the sample was not nationally representative to some extent, all patients were from all over the country towards these two hospitals (tertiary referral hospitals).

However, despite these strengths, some limitations should be noted during interpretation and policy formulation. First of all, this study was a cross-sectional study, which could only provide clues to the etiology, and further exploration was needed to prove the causal relationship; Secondly, our study used secondary data and as such, we were unable to measure quantities and type of alcohol and tobacco consumed as well as obtain information on physical activities which have been found to be associated with hypertension. Lastly, the sample size was relatively not large and caution should be taken when generalizing findings on high blood pressure as data used were only reported from two hospitals.

To validate findings, additional studies should be conducted in other hospitals in the country and take into others characteristics including more biomarkers. Score based models and nomograms should be used to support clinical implementation of risk models. A random effect logistic regression or Bayesian regression based on Markov chain Hamiltonian Monte Carlo simulations and Langevin algorithms could give precision in the estimation of model’s parameters. Bayesian credibility intervals as such these methods are recommended for future research. The main interest of this study was to identify predictive risk factors of hypertension which allowed prediction of hypertension’s occurrence controlling possible cofounders.

## Conclusion

This study showed that the hypertension prevalence was relatively high. Hypertension’s prevalence was not significantly different in men and women. Predictive risk factors of hypertension were advanced age smoking, presence of chronic kidney failure, existing cardiovascular impairment, educational level and body mass index.

The lowest predicted probability of hypertension was observed in young patients with no risk factors. High predicted probabilities to become hypertensive were observed in patients with coexistence of two or more risk factors. Resources in Burundi are scare, therefore, the tackling the high burden of cardiovascular diseases should be based on instituting systems for early detection and prompt treatment especially those identified as high risks.

To our knowledge, no study combining the predictive risk factors analysis and probabilities predictions have been carried in Burundi. At the community level, efforts should be channelled towards intensifying innovative and inclusive health promotion aimed at behaviour change. At the health system, creating a risk-based nomogram based on these identified risks factors could allow those at high risks to be identified early and well-targeted with the needed treatment.

Finally, provision of long term care for those identified cases will depend on not just consistent treatment but also on the overall health systems’ strengthening. This will ensure sustainability and effectiveness of public health interventions aimed at chronic diseases tackling along with other high burden infectious diseases.

## Supporting information

S1 Data (SAV)

## List of abbreviations

AIC: Akaike Information Criterion
AOR: Adjusted Odds Ratio
AUC: Area Under Curve
BMI: Body Mass Index
BP: Blood Pressure
CI: Confidence Interval
CKF: Chronic Kidney failure
Df: Degrees of freedom
CS: Clinical symptoms
FHH: Family History with Hypertension
HTN: Hypertension
H-: Normotensive people
H+: Hypertensive people
OR: Odds Ratio
ROC: Receiver Operating Characteristic
SSA: Sub-Saharan Africa
WHO: World Health Organisation

## References

[1] Mills K.T., Stefanescu A., and He J., The global epidemiology of hypertension. Nature Reviews Nephrology, 2020. 16(4): p. 223–237. doi: 10.1038/s41581-019-0244-2 32024986 PMC7998524

[2] Umashankar A. and Prabhu P., Hearing Loss and Hypertension: A Literature Review. Indian Journal of Otolaryngology and Head & Neck Surgery, 2021: p. 1–9. doi: 10.1007/s12070-010-0048-y 36032913 PMC9411486

[3] Egbi O.G., Ahmed S.D., and Madubuko R., Prevalence and biosocial determinants of hypertension in a rural population in Edo State, Southern Nigeria. African Journal of Primary Health Care & Family Medicine, 2021. 13(1): p. 7. 10.4102/phcfm.v13i1.2833 PMC8335784 34212744

[4] Wang C., et al., Bioinspired therapeutic platform based on extracellular vesicles for prevention of arterial wall remodeling in hypertension. Bioactive Materials, 2021. doi: 10.1016/j.bioactmat.2021.06.005 34541415 PMC8427223

[5] Liu W., et al., Uncontrolled hypertension associates with subclinical cerebrovascular health globally: a multimodal imaging study. European Radiology, 2021. 31(4): p. 2233–2241. doi: 10.1007/s00330-020-07218-5 32929643

[6] Wang C., et al., Association of age of onset of hypertension with cardiovascular diseases and mortality. Journal of the American College of Cardiology, 2020. 75(23): p. 2921–2930. doi: 10.1016/j.jacc.2020.04.038 32527401

[7] Kidoguchi S., et al., New concept of onco-hypertension and future perspectives. Hypertension, 2021. 77(1): p. 16–27. doi: 10.1161/HYPERTENSIONAHA.120.16044 33222548

[8] Rudd K.E., et al., Global, regional, and national sepsis incidence and mortality, 1990–2017: analysis for the Global Burden of Disease Study. The Lancet, 2020. 395(10219): p. 200–211. 10.1016/S0140-6736(19)32989-7 PMC6970225 31954465

[9] Villarreal-Zegarra D., Carrillo-Larco R.M., and Bernabe-Ortiz A., Short-term trends in the prevalence, awareness, treatment, and control of arterial hypertension in Peru. Journal of human hypertension, 2021. 35(5): p. 462–471. doi: 10.1038/s41371-020-0361-1 32518303 PMC8134053

[10] Lin X., et al., Global, regional, and national burden and trend of diabetes in 195 countries and territories: an analysis from 1990 to 2025. Scientific reports, 2020. 10(1): p. 1–11. doi: 10.1038/s41598-019-56847-4 32901098 PMC7478957

[11] Rethy L., et al., Trends in hypertension-related cardiovascular mortality in the United States, 2000 to 2018. Hypertension, 2020. 76(3): p. e23–e25. doi: 10.1161/HYPERTENSIONAHA.120.15153 32654559 PMC9390965

[12] Sayed M.A., et al., Stroke promotes the development of brain atrophy and delayed cell death in hypertensive rats. Scientific reports, 2020. 10(1): p. 1–14. doi: 10.1038/s41598-019-56847-4 33214598 PMC7678843

[13] Zhang X., et al., Catastrophic health expenditure: a comparative study between hypertensive patients with and without complication in rural Shandong, China. BMC Public Health, 2020. 20(1): p. 1–13. doi: 10.1186/s12889-019-7969-5 32321485 PMC7178564

[14] Londoño Agudelo E., et al., Out-of-pocket expenditure for hypertension care: a population-based study in low-income urban Medellin, Colombia. Global Health Action, 2020. 13(1): p. 1806527. doi: 10.1080/16549716.2020.1806527 32867605 PMC7480425

[15] Božič Ješe N., et al., May Measurement Month 2019: an analysis of blood pressure screening results from Slovenia. European Heart Journal Supplements, 2021. 23(Supplement_B): p. B131–B133. 34733127 10.1093/eurheartj/suab030 PMC8559328

[16] Idris A. and Olaniyi O., ANALYSIS OF ECONOMIC BURDEN OF HYPERTENSION IN NIGERIA. Nigerian Journal of Economic and Social Studies, 2020. 62(2).

[17] Hu L., et al., Prevalence and Risk Factors of Prehypertension and Hypertension in Southern China. PLoS One, 2017. 12(1): p. e0170238. doi: 10.1371/journal.pone.0170238 28095471 PMC5240940

[18] HAMET P., AMBOURHOUET A.-M.A., and MBENZA B.L., CHAPITRE 6 L’hypertension artérielle. SANTÉ MONDIALE, 2020.

[19] Vardell E., Global health observatory data repository. Medical reference services quarterly, 2020. 39(1): p. 67–74. doi: 10.1080/02763869.2019.1693231 32069199

[20] Ahmed S., et al., Inequalities in the prevalence of undiagnosed hypertension among Bangladeshi adults: evidence from a nationwide survey. International journal for equity in health, 2019. 18(1): p. 1–12. 10.1186/s12939-019-0930-5 PMC6377713 30770739

[21] Hasan M.M., et al., Examining the prevalence, correlates and inequalities of undiagnosed hypertension in Nepal: a population-based cross-sectional study. BMJ open, 2020. 10(10): p. e037592. doi: 10.1136/bmjopen-2020-037592 33004393 PMC7534699

[22] Nahimana M.-R., et al., A population-based national estimate of the prevalence and risk factors associated with hypertension in Rwanda: implications for prevention and control. BMC public health, 2018. 18(1): p. 1–11. 10.1186/s12889-017-4536-9 PMC5504833 28693458

[23] Mohamed S.F., et al., Uncontrolled hypertension among patients with comorbidities in sub-Saharan Africa: protocol for a systematic review and meta-analysis. Systematic reviews, 2020. 9(1): p. 1–5. 31948464 10.1186/s13643-020-1270-7 PMC6964205

[24] Chikowore T., et al., Non-communicable diseases pandemic and precision medicine: Is Africa ready? EBioMedicine, 2021. 65: p. 103260. doi: 10.1016/j.ebiom.2021.103260 33639396 PMC7921515

[25] Mash R. and Schouw D.D., Cost and consequence analysis of the Healthy Choices at Work programme to prevent non-communicable diseases in a commercial power plant, South Africa. African Journal of Primary Health Care and Family Medicine, 2020. 12(1): p. 1–8. 10.4102/phcfm.v12i1.2217 PMC7343951 32634016

[26] Bertram M.Y., et al., Cost-effectiveness of population level and individual level interventions to combat non-communicable disease in Eastern Sub-Saharan Africa and South East Asia: a WHO-CHOICE analysis. International Journal of Health Policy and Management, 2021. doi: 10.34172/ijhpm.2021.37 34273918 PMC9278376

[27] Mogas S.B., et al., Burden of Undiagnosed Hypertension among Adults in Urban Communities of Southwest Ethiopia. Integrated Blood Pressure Control, 2021. 14: p. 69. doi: 10.2147/IBPC.S293251 34054307 PMC8153066

[28] Osei E., et al., Mapping evidence of mobile health technologies for disease diagnosis and treatment support by health workers in sub-Saharan Africa: a scoping review. BMC Medical Informatics and Decision Making, 2021. 21(1): p. 1–18. doi: 10.1186/s12911-020-01362-0 33407438 PMC7789784

[29] Cao C., et al., Prehypertension during pregnancy and risk of small for gestational age: a systematic review and meta-analysis. The Journal of Maternal-Fetal & Neonatal Medicine, 2020. 33(8): p. 1447–1454. doi: 10.1080/14767058.2018.1519015 30173597

[30] Kamble T., Kashid D., and Sakate D., Consistent and robust variable selection in regression based on Wald test. Communications in Statistics-Theory and Methods, 2019. 48(8): p. 1981–2000.

[31] Falk C.F. and Muthukrishna M., Parsimony in model selection: Tools for assessing fit propensity. Psychological Methods, 2021. doi: 10.1037/met0000422 34647757

[32] Josephat P. and Ame A., Effect of testing logistic regression assumptions on the improvement of the propensity scores. Int. J. Stat. Appl, 2018. 8: p. 9–17.

[33] Team, R.C., R: A language and environment for statistical computing. 2013.

[34] Williams B., et al., 2018 ESC/ESH Guidelines for the management of arterial hypertension: The Task Force for the management of arterial hypertension of the European Society of Cardiology (ESC) and the European Society of Hypertension (ESH). European heart journal, 2018. 39(33): p. 3021–3104. doi: 10.1093/eurheartj/ehy339 30165516

[35] Ostchega Y., et al., Hypertension prevalence among adults aged 18 and over: United States, 2017–2018. 2020. 32487290

[36] Samanic C.M., et al., Prevalence of self-reported hypertension and antihypertensive medication use among adults—United States, 2017. Morbidity and Mortality Weekly Report, 2020. 69(14): p. 393. doi: 10.15585/mmwr.mm6914a1 32271727 PMC7147902

[37] Desormais I., et al., The prevalence, awareness, management and control of hypertension in men and women in Benin, West Africa: the TAHES study. BMC cardiovascular disorders, 2019. 19(1): p. 1–12. doi: 10.1186/s12872-018-0984-0 31881946 PMC6933658

[38] Omar S.M., et al., Prevalence and associated factors of hypertension among adults in Gadarif in eastern Sudan: a community-based study. BMC public health, 2020. 20(1): p. 1–6. doi: 10.1186/s12889-019-7969-5 32138715 PMC7059678

[39] Lavôr L.C.d.C, et al., Prevalence of arterial hypertension and associated factors: a population-based study. Revista da Associação Médica Brasileira, 2020. 66: p. 630–636. doi: 10.1590/1806-9282.66.5.630 32638969

[40] Price A.J., et al., Prevalence of obesity, hypertension, and diabetes, and cascade of care in sub-Saharan Africa: a cross-sectional, population-based study in rural and urban Malawi. The lancet Diabetes & endocrinology, 2018. 6(3): p. 208–222. 10.1016/S2213-8587(17)30432-1 PMC5835666 29371076

[41] Rahman M.N., et al., Prevalence of Hypertension and Its Associated Factors among Adults in Selected Areas of Bangladesh: A Community Based Cross-sectional Study. American Journal of Hypertension, 2021. 8(1): p. 1–7.

[42] Yaya S., et al., Prevalence and Socioeconomic Factors of Diabetes and High Blood Pressure Among Women in Kenya: A Cross-Sectional Study. Journal of Epidemiology and Global Health, 2021: p. 1–8. doi: 10.2991/jegh.k.201028.001 34734380 PMC8664325

[43] Sapkota A., et al., Prevalence and associated factors of hypertension among veterans of the Indian Gorkha regiments living in Pokhara Metropolitan City, Nepal. BMC Health Services Research, 2021. 21(1): p. 1–6. doi: 10.1186/s12913-020-05996-8 34470648 PMC8408920

[44] Roba H.S., et al., Prevalence of hypertension and associated factors in Dire Dawa city, Eastern Ethiopia: a community-based cross-sectional study. International journal of hypertension, 2019. 2019. doi: 10.1155/2019/9878437 31223500 PMC6541960

