# Supplementary material for: Prevalence and predictive risk factors of hypertension in patients hospitalized in Kamenge Military hospital and Kamenge University teaching hospital in 2019: A fixed effect modelling study in Burundi
Source: PLoS One. 2021 Dec 13;16(12):e0260225. doi: 10.1371/journal.pone.0260225 (PMC8668094; doi:10.1371/journal.pone.0260225)
Supplement: Attachment — Submitted filename: Response to Reviewers1.docx [file pone.0260225.s002.docx]

**The Editor,**

**PLOS ONE,**

Comments and their respective responses

***Reviewer 1***

**Comment 1**: The attached database has 304 respondents, while the manuscript shows 353. It is not possible to repeat the analyzes listed in the paper. A proper database needs to be attached.

**Response 1**: Thank you for the comment. A dataset of 353 respondents have been attached.

**Comment 2:** The population of respondents is not precisely explained through the structure of wards and patients in hospitals. What are the primary diagnoses for which they were admitted to the hospitals? How was the randomization done in relation to the hospital ward?

**Response 2:** Thank you for the comments. Description have been made in the manuscript

**Comment 3:** When calculating the sample size, the prevalence of hypertension was taken to be 0.5, which is a large value.

**Response 3:** Yet, studies to understand the epidemiology and associated in the context of Burundi are lacking. Without prevalence of hypertension in previous studies conducted in Burundi, we used p=0.5 as recommended in literature on sampling method (**Giezendanner, François Daniel. "Taille d’un échantillon aléatoire et marge d’erreur." *Instruction Publique, Culture et Sport* (2012): 7.)** .

**Comment 4:** The incidence of Chronic kidney failure is 52.1% (184/353 * 100), which is a high incidence. Additionally, whether chronic kidney failure is a predictor or a consequence of hypertension. Can these patients be regarded as predictors of hypertension in the study?

**Response 4:** This incidence is relatively high because the study included **all patients from intensive care unit with chronic kidney failure**. Majority of these patients are hospitalized there because this areas are located together with haemodialysis unit. Therefore, these patients can be regarded as predictors as the **Renin-Angiotensin-Aldosterone System** participate in the development of hypertension from chronic kidney failure.

**Comment 5:** It is not explained how and based on what data was the General cardiovascular risk calculated and what it refers to.

**Response 5:** The cardiovascular risk was based on both blood pressure level, risk factors and clinical symptoms. The increase of blood pressure level associated with increase of risk factors (them founded among our patients) increase the risk. In the data set, we scored each risk factors by one 1 point. For each patients, calculated the total of the points and we classified it in four classes: Zero risk factor,1-2 Risk factors, 3 and more risk factors and $\geq$ 3 risk factors associated with clinical blood pressure.

***Therefore, as this evaluation is not linked with title and specific objectives of the study, the table 5 have been removed in the manuscript.***

**Comment 6:** More than 50% of references are older than 5 years. There are newer publications for this topic, so I suggest adding them.

**Response 6:** New publications are added.

**Comment 7:** How the results obtained on the basis of hospital patients can be generalized to the population of Burundi?

**Response 7:** Partially, we generalized these findings because these hospitals are the tertiary national reference hospitals which receive patients from across the country. On other side, these findings could not be generalized because the sample is not nationally representative. We highlighted it is as limit of the study, prompt to conduct others study on the national level. In conclusion, these findings cannot be generalized and suggest to conduct another study in most of Burundian hospitals.

**Comment 8:** Include BMI in the model as an ordinal variable with all categories or compare Overweight and Obesity in relation to Underweight and Normal.

**Response 8:** BMI have been included in the model. It has been categorized as suggested

Comment 9: Is cardiovascular comorbidity a predictor or a consequence of hypertension?

Response 9: By mention of cardiovascular comorbidities, we implied the presence of an existing cardiovascular impairment apart from hypertension. We have now clarified that throughout the manuscript. Thank you.

Comment 10: In logistic regression, the odds ratio is obtained, so the results should be interpreted in that way.

Response 10: Results have been presented using odds ratios and adjusted odds across the manuscript. Interpretation follows same

Comment 11: The paper does not state the level of significance.

Response 11:	Level of significance has been stated on page 6 line 7.

Comment 12: Delete AIC values from tables with univariate analyzes because they do not contribute to informativeness.

Response 12: Changes have been made in the manuscript

Comment 13: In the attached database it is necessary to check the values for: age (range 15-107 years) and BMI (minimum value is 6.72)

Response 13: These values are correct even if they seems to outliers.

Comment 14: In Table 3 the p-value for Smoking is 0.081, which is not statistically significant.

Response 14: Even if the table 3 have been removed in manuscript, the selection of significant variables was based on AIC and the best model is the one with low AIC. As we used the backward method, when we remove tobacco consumption, the AIC increases which is not recommended in modelling. Beside the Akaike criterion, the model without tobacco decrease the AUC, and consequently the predictive power. All these augments allowed us to include tobacco use in the model.

**Comment 15**: In Table 1 the sum of all categories in column N is 353. In cell named Total, the value is 309.

**Response15:** Changes have been made in the manuscript

**Comment 16:** All numeric values in tables and text should be set to one decimal place.

**Response 16:** Changes have been made in the manuscript

***Reviewer 2***

The topic of the paper is interesting, always actual and always need new information-up to date. The topic is also relevant in the application of appropriate statistical modelling to identify risk factors.

**Comment 1:** In order for the paper to be publish, it is necessary to change the way of describing the used statistical methods and presenting the results. In the material and methods, it is described how the required sample size was calculated. It only needs to be describe, without writing formulas.

**Response 1:** Thank you for the comment. All those changes have been made in the manuscript.

**Comment 2:** In the part of statistical analysis, it should be described why certain statistical tests and analyzes were used. Describe the tests used in the different data collected and analyzed

**Response 2:** Changes have been made in the manuscript

**Comment 3:**  It is not necessary to show the formulas of the regression analysis used. This is unnecessary if the topic of the paper is hypertension in a certain patient population.

**Response 3:** Changes have been made in the manuscript

**Comment 4:** This way of writing the methodology as well as presenting the results is matching with the paper whose topic is logistic regression analysis, with example of a study to identify risk factors for the occurrence of hypertension in the observed patient population.

**Response 4:** changes have been made in the manuscript

**Comment 5:** Table 1: What 95% CI refers.

**Response 5:** 95% CI refers to proportion hypertension ‘’P+ ‘’

**Comment 6:** Table 2: explain what AIC is.

**Response 6 :** AIC have been removed in the Table 3 but is very well explained in manuscript as Akaike Information Criterion used to choose best model.

**Comment 7:** Is table 3 required?

**Response 7:** No it is not required and we removed it in the manuscript

**Comment 8:** Fig. 1 and Fig. 2, and Fig. 4 are unclear. Describe the adequacy of the model, and the tests used for testing in the methodology.

**Response 8**: Changes have been made in the manuscript. The adequacy of the model is described and the Hosmer-Lemeshow have been used to test it.

Kind regards,

Arnaud IRADUKUNDA (Corresponding author)
