# Supplementary material for: Prevalence and predictive risk factors of hypertension in patients hospitalized in Kamenge Military hospital and Kamenge University teaching hospital in 2019: A fixed effect modelling study in Burundi
Source: PLoS One. 2021 Dec 13;16(12):e0260225. doi: 10.1371/journal.pone.0260225 (PMC8668094; doi:10.1371/journal.pone.0260225)
Supplement: Attachment — Submitted filename: Response to Reviewers1.docx [file pone.0260225.s003.docx]

**The Editor,**

**PLOS ONE,**

**Journal Requirements:**

Please review your reference list to ensure that it is complete and correct. If you have cited papers that have been retracted, please include the rationale for doing so in the manuscript text, or remove these references and replace them with relevant current references. Any changes to the reference list should be mentioned in the rebuttal letter that accompanies your revised manuscript. If you need to cite a retracted article, indicate the article’s retracted status in the References list and also include a citation and full reference for the retraction notice.

Response: We confirm the reference list is complete and accurately reflects all the in-text citations. There are no cited papers which have been retracted and the list as it stands need no further changes. Thank you.

**Reviewer comments and their respective responses**

Reviewer #1: ***I suggest that the manuscript can be accepted for publication after a minor revision:***

**Comment 1**. The attached database does not have a variable BMI divided to categories (Underweight and healthy, Overweight, Obese)

**Response:** Thank you for the comment. As we used R software, we coded directly the BMI’s categories in R: These are the code used to carry out the BMI and its categories:

a. BMI<-(WEIGHT/ (HEIGHT^2)) #Body mass index calculation

b. BMI cat<- ifelse (BMI<25,"Underweight/Normal", (BMI<30,"Overweight", "Obesity")) #Body mass index categorization.

Coment.2. Check all p-values (for example, the p-value for EL in Table 2 is different from the p-value obtained after analysis from the database)

Response 2: Thank you for your comments. All p value were have been well checked.

Comment 3. Check and correct all errors in the Tables (one of the examples [2.1-12.4-11.15])

**Response 3**: Thank you for the comment. Change have been made in the main manuscript

Comment. 4. Equalize decimal places for numbers – number of decimal places should be consistent throughout the paper

Response 4: Changes are made in the main manuscript

Comment. 5. Correct typing mistakes, sometimes there is no break, and sometimes it is in the wrong place.

Response 5: Changes are made in the main manuscript

Reviewer #2: (No Response)

Kind regards,

Arnaud IRADUKUNDA (Corresponding author)
